# Role of Vitamin B12 and Folate in Metabolic Syndrome

**DOI:** 10.7759/cureus.18521

**Published:** 2021-10-06

**Authors:** Tejaswini Ashok, Harivarsha Puttam, Victoria Clarice A Tarnate, Sharan Jhaveri, Chaithanya Avanthika, Amanda Guadalupe Trejo Treviño, Sandeep SL, Nazia T Ahmed

**Affiliations:** 1 Internal Medicine, Jagadguru Sri Shivarathreeshwara Medical College, Mysore, IND; 2 Internal Medicine, Employees' State Insurance Corporation Medical College and Hospital, Hyderabad, IND; 3 Medicine, Far Eastern University - Nicanor Reyes Medical Foundation, Quezon City, PHL; 4 Internal Medicine, Smt. Nathiba Hargovandas Lakhmichand Municipal Medical College, Ahmedabad, IND; 5 Medicine and Surgery, Karnataka Institute of Medical Sciences, Hubli, IND; 6 Pediatrics, Karnataka Institute of Medical Sciences, Hubli, IND; 7 Medicine, Facultad de Medicina Universidad Autonoma de Nuevo León, Monterrey, MEX; 8 Internal Medicine, SRM Medical College Hospital & Research Centre, Kattankulathur, IND; 9 Medicine, Shahabuddin Medical College and Hospital, Dhaka, BGD

**Keywords:** insulin resistance, cardiovascular disease, nutrition and metabolism, obesity and diabetes, vitamin b supplementation, folic acid supplementation, vitamin b12 supplementation, diabetes and metabolic syndrome

## Abstract

Metabolic syndrome (MS) is a collection of pathological metabolic conditions that includes insulin resistance, central or abdominal obesity, dyslipidemia, and hypertension. It affects large populations worldwide, and its prevalence is rising exponentially. There is no specific mechanism that leads to the development of MS. Proposed hypotheses range from visceral adiposity being a key factor to an increase in very-low-density lipoprotein and fatty acid synthesis as the primary cause of MS. Numerous pharmaceutical therapies are widely available in the market for the treatment of the individual components of MS. The relationship between MS and vitamin B complex supplementation, specifically folic acid and vitamin B12, has been a subject of investigation worldwide, with several trials reporting a positive impact with vitamin supplementation on MS.

In this study, an all-language literature search was conducted on Medline, Cochrane, Embase, and Google Scholar till September 2021. The following search strings and Medical Subject Headings (MeSH) terms were used: “Vitamin B12,” “Folate,” “Metabolic Syndrome,” and “Insulin Resistance.” We explored the literature on MS for its epidemiology, pathophysiology, newer treatment options, with a special focus on the effectiveness of supplementation with vitamins B9 and B12.

According to the literature, vitamin B12 and folate supplementation, along with a host of novel therapies, has a considerable positive impact on MS. These findings must be kept in mind while designing newer treatment protocols in the future.

## Introduction and background

Metabolic syndrome (MS) is one of the most debilitating disorders currently affecting large populations worldwide, with its prevalence rising exponentially. MS refers to a group of pathological metabolic conditions including insulin resistance, central or abdominal obesity, atherogenic dyslipidemia, and hypertension [1]. According to the Adult Treatment Panel III (ATP III) classification, the parameters of MS include waist circumference of >102 cm for men and >88 cm for women, fasting blood glucose of >6.1 mmol/L, blood pressure of >135/85 mmHg, triglyceride level of >1.7 mmol/L, and high-density lipoprotein-cholesterol (HDL-C) level of <1.0 mmol/L [2]. The diagnosis of MS is established if an individual meets three of these five parameters [2]. MS, also known as insulin resistance syndrome, syndrome X, as well as the deadly quartet, has been recognized as an important risk factor for the development of cardiovascular disease and type 2 diabetes mellitus, with an estimated two-fold and five-fold increased incidence risk, respectively [3]. By 2030, approximately 38% of the adult population globally is predicted to be overweight and roughly 20% to be obese, which increases the prevalence of morbidity caused by chronic diseases [4]. Numerous pharmaceutical therapies are widely available in the market for the treatment of the individual components of MS, such as statins, antihypertensives, and antidiabetic drugs. Meanwhile, suggested dietary supplements are also available, such as folic acid and vitamin B12, which may be beneficial in the treatment of MS.

Vitamins B9 (folate) and B12 (cobalamin) are essential water-soluble vitamins that serve an important role in DNA methylation and the homeostasis of both amino acids and lipids through the regulation of one-carbon metabolism [5]. One-carbon metabolism is a chain of interconnected biochemical pathways mainly powered by folate and methionine to generate methyl donors utilized by several cellular processes [5]. Because vitamins B9 and B12 are crucial for cell metabolism, their deficiency can lead to alarming health consequences.

Low levels of folate and cobalamin can cause DNA synthesis interference, cellular inflammation, and elevated synthesis of fat and homocysteine [6]. Homocysteine is a non-proteinogenic amino acid produced by the breakdown of dietary proteins such as methionine [7]. Hyperhomocysteinemia can contribute to the development of cardiovascular and cerebrovascular diseases due to atherosclerotic vascular-endothelial injury [8], as well as the development of insulin resistance [9]. Studies have shown that folic acid, vitamin B12, and other B-complex supplements are effective in reducing homocysteine levels in the plasma [10], thus leading to the reduction of the prevalence of MS.

The relationship between MS and vitamin B complex, specifically folic acid and vitamin B12, has been the subject of many studies worldwide. Several studies have reported a positive association between the supplementation of folic acid and vitamin B12 and their effectiveness in the reduction of cardiovascular disease [11] and insulin resistance [12]. The scope of this literature review is to study and analyze scientific articles from PubMed and other academic journal libraries regarding the possible role and effectiveness of folic acid and vitamin B12 in MS patients. By reviewing the efficacy of folic acid and vitamin B12 supplementation in MS, we aim to enumerate potential recommendations which can be added to the current treatment regimen for MS patients. However, considerations of additional studies, such as those relating to the appropriate frequency, duration, and dosage of folic acid and vitamin B12 as an adjunct for MS treatment, will need to be evaluated to prevent any unwanted adverse effects.

## Review

Sources, metabolism, and interactions

Sources

It is important to shed light on the dietary sources of folic acid and cobalamin, being vitamins of public health significance. Excellent sources of vitamin B9 (folate) are green leafy vegetables, citrus fruits, pulses, cereals, liver, and egg yolk while vitamin B12 is majorly present in meat, eggs, milk, dairy products, fish, and shellfish, with the concentration of cobalamin varying within the food of ruminant origin with the highest concentrations found in offal such as liver and kidney. Various studies have been conducted to measure the adequacy of dietary intake of folic acid and cobalamin in different populations owing to its key function in the one-carbon metabolism and its clinical significance [13,14]. In Europe, a study demonstrated subclinical deficiency of vitamin B9 and B12 to be prevalent in 20% of adolescents [15]. Correspondingly, nutritional data surveys conducted in cross-country comparative studies across the European continent showed a mean prevalence of inadequacy at or below 10% for the elderly and below 20% for the adult population [16]. Remarkable differences have been noted in vitamin B12 levels in cohort studies of meat eaters, fish eaters, vegetarians, and vegans, highlighting the inadequate dietary intake among vegans and vegetarians [17,18]. Attempts have been made to identify naturally occurring cobalamin-rich, plant-derived foods, such as dried shiitake mushroom fruiting bodies, dried green laver (*Enteromorpha species*) and purple laver (*Porphyra* species), aquatic plant *Wolffia globosa* (Mankai), sea buckthorn (*Hippophae rhamnoides*) berries and granulate products, and sidea couch grass (*Elymus repens*) products (dry extract and ground) [19-22]. A clinical trial conducted in India to determine improvement in vitamin B12 status among deficient vegetarians with dairy cow’s milk intake concluded that it can drastically improve blood cobalamin levels [23].

Finding new methods that increase cobalamin content in vegetarian diets has been an important field of research. Two such effective methods are the addition of cow manure to significantly increase the vitamin B12 content of spinach leaves and the fermentation of food with lactic acid or propionic bacteria [19]. Another strategy aimed to enrich animal products with natural folic acid through the addition of high doses of synthetic folic acid to animal feed. In another study, supplementation of hen’s diet with folate enriched hen’s eggs to a maximum folate content of approximately 2.5 times that of a normal egg [24]. Increasing fiber concentration of cow’s diet was positively associated with vitamin B12 concentration in milk, whereas a negative relationship was established between folate and dietary fiber concentrations [25,26]. Furthermore, intramuscular injection of vitamin B12 in early-lactation dairy cows increased vitamin B12 content in milk [27].

The association of impaired folate levels with various pathological consequences led to the initiation of exogenous supplementation and biofortification approaches. Interventional trials aimed at proving the linkage of maternal vitamin B12 consumption with the concentration of vitamin B12 in breast milk showed that vitamin B12 concentration in human milk can be modified via supplementation during periconceptional and lactational periods [28]. Implementation of mandatory fortification of cereal grains with vitamin B12 was attempted in 1998 in the United States, followed by several other countries in subsequent years. However, despite evidence of the benefits of fortification, concern has been raised regarding the detrimental effects of exposure to synthetic or crystalline folic acid, the most important being the masking of vitamin B12 deficiency and its progression leading to irreversible neurological deficits [29].

For the benefit of vulnerable populations, a holistic approach of triple fortification (iron, iodine, folic acid) and quadruple fortification (iron, iodine, folic acid, vitamin B12) has been undertaken recently [30,31]. With increasing attention on the nutritional benefits of insects, the folic acid content in two cricket species, namely, *Scapsipedus icipe* and *Gryllus bimaculatus*, was measured and found to be much higher than animal and plant-based food sources, making crickets a cheaper alternative as opposed to commercial supplements. This demonstrated the need for the inclusion of crickets into the human diet, especially in resource-poor populations [32]. In a study directed at isolating strains of lactobacilli synthesizing the highest folate concentration by milk fermentation, it was found that *Lactobacillus fermentum* (from kefir granules), *Lactobacillus plantarum* (from salted mustard), and *Lactobacillus rhamnosus* (from breast milk) produced the highest concentrations of folic acid in the absence of pre-existing folate in milk [33]. An interventional study conducted in India to estimate the potentiality of tea as a scalable vehicle for fortification with vitamins B9 and B12 successfully showed that the daily consumption of a cup of vitamin-fortified tea for two months could benefit women of reproductive age [34]. Investigations on the dietary value of freshwater living green unicellular algae *Chlorella* species proved the presence of large amounts of folate. Moreover, a meta-analysis on the effect of its supplementation on cardiovascular risk factors showed that it improves total cholesterol levels, low-density lipoprotein-cholesterol (LDL-C) levels, systolic blood pressure, diastolic blood pressure, and fasting blood glucose levels [35].

With the advent of metabolic engineering, new studies are being conducted to assist in the construction of microbial cell factories for large-scale vitamin B12 production [36]. All these studies have the common goal of finding ways to improve folate and cobalamin levels in the general population, thus emphasizing the importance of these vitamins in the prevention of avoidable hematological and neurological complications.

Metabolism

Understanding the metabolism and the effects of alterations in each of its steps is of utmost importance as it would help us interpret and prognosticate the pathological outcomes and therapeutic possibilities. The metabolic processes of vitamins B9 and B12 are complex and interrelated at the cellular level. Following the breakdown of cobalamin, its reduced form is directed toward the two enzymes, namely, cytosolic methionine synthase and mitochondrial methylmalonyl-coenzyme A mutase (MUT) [37]. MUT utilizes an adenylated form of cobalamin in the catabolism of branched-chain amino acids, odd-chain fatty acids, and the side chain of cholesterol, whereas methionine synthase requires a methylated form of cobalamin and catalyzes the remethylation of homocysteine to methionine (using 5-methyltetrahydrofolate [CH3-THF] as a methyl donor) [37]. The methionine produced is further converted to S-adenosyl methionine (AdoMet, often called SAM), whose methyl group can be further donated to essentially important methylated compounds such as creatine, epinephrine, sarcosine, methylated DNA, RNA, and proteins [37].

The linkage of cobalamin metabolism with the folate-mediated one-carbon metabolism is attributed to the utilization of CH3-THF as the methyl donor [37]. CH3-THF is the most common form of folate in blood and tissues [38]. Depending on the cellular requirements, the folate-mediated one-carbon metabolic pathway helps folate to interchange between its different forms.

This metabolic pathway is mainly compartmentalized between cytosol and mitochondria depending on the mono/polyglutamate tail of folate which serves as a localization signal within the cell [39]. Even though two separated parallel pathways exist in cytosol and mitochondria, mitochondrial one-carbon oxidation accounts for approximately 50% of the nicotinamide adenine dinucleotide phosphate produced in the cell [40]. The deficiency of vitamin B12 and/or folate leads to the disruption of the one-carbon cycle and its downstream metabolic processes. Therefore, a deeper understanding of the cause and effect of the one-carbon metabolism is essential to obtain considerable insights into its role in the development of metabolic diseases [41].

Interactions

Mammalian cells exhibit two different vitamin B12-dependent reactions: conversion of methylmalonyl coenzyme A to succinyl coenzyme A by the enzyme MUT and the methylation of homocysteine to methionine by methionine synthase. The deficiency of vitamin B12 results in elevated levels of homocysteine and methylmalonic acid in the circulation. These function as biochemical markers of vitamin B12 status. Studies have proven that excess folate resulting mostly due to high folic acid intake interferes with cobalamin metabolism and worsens conditions associated with vitamin B12 insufficiency [42].

Malabsorption of vitamin B12 among the elderly has been observed commonly due to gastric atrophy or as a result of *Helicobacter pylori* infection. Pernicious anemia due to lack of gastric acid and intrinsic factors is another cause of B12 malabsorption. Low gastrointestinal motility resulting from gastrointestinal surgery, including gastric bypass, jejunal diverticulosis, blind-loop syndrome after intestinal surgery, and structural abnormalities such as diverticulosis in the duodenum or jejunum lead to bacterial overgrowth in the upper intestine, which, in turn, impairs the absorption of vitamin B12 from food. The proposed mechanisms for this include competition with intestinal bacteria for the uptake of vitamin B12, bacterial conversion of the vitamin to inactive analogs, and hypochlorhydria [43,44]. Some of the infections interfering with vitamin B12 absorption are fish tapeworm infection,* Giardia lamblia* infection, malaria, human immunodeficiency virus infection, and acquired immunodeficiency syndrome [44]. Tropical sprue, celiac disease, Zollinger-Ellison syndrome, Imerslund-Gräsbeck syndrome, juvenile pernicious anemia, pancreatic insufficiency, and defects in haptocorrin binding proteins are some of the pathological conditions associated with reduced cobalamin absorption [44]. Chronic pancreatitis and cystic fibrosis result in impaired trypsin secretion which is required for the release of vitamin B12 from transcobalamin-1 in the intestine [44].

Drugs interacting with vitamin B12 absorption include histamine receptor antagonists such as famotidine and proton-pump inhibitors such as omeprazole and lansoprazole. Cimetidine has been shown to inhibit the secretion of gastric acid, pepsin, and intrinsic factors [45-47]. A dose-related effect of cimetidine on vitamin B12 absorption was observed with intakes >1,000 mg/day reducing food-bound vitamin B12 absorption, but not 400 mg/day [45,48]. Similarly, a 20 mg/day dose of omeprazole reduced vitamin B12 absorption from a protein-bound source by approximately 70%, while 40 mg/day reduced absorption by approximately 90% [49].

Moreover, mild transcobalamin I deficiency has been linked to the risk of low serum cobalamin concentration. A common gene polymorphism was observed in vitamin B12 carrier protein, transcobalamin II, where proline (P) is substituted for arginine (A), affecting the mean total plasma concentrations of vitamin B12 and homocysteine [50]. Unlike folic acid, dietary folates are relatively unstable to oxidation and heat; hence, large losses can occur during food preparation and cooking. Boiling has been shown to destroy up to 80% of folate in green vegetables, whereas chopping and grinding spinach has been shown to increase folate bioavailability [51,52]. Furthermore, ascorbic acid in foods was shown to improve folate stability [53].

Chronic alcoholics are prone to vitamin B9 deficiency due to low folate intake, poor folate absorption due to impaired transcription of the intestinal folate carrier, reduced liver uptake and storage, and increased urinary excretion [54]. The speed of onset of alcoholic liver disease was assessed in a micro-pig model, which demonstrated that the onset was more rapid when the animals were provided with a folate-deficient diet than when folate intake was adequate [55]. Medications that impair folate status include methotrexate (a folate antagonist used in the treatment of rheumatoid arthritis, inflammatory bowel disease, etc.), anticonvulsants (phenytoin, phenobarbital, and Dilantin), sulfasalazine (for the treatment of chronic ulcerative colitis), and pyrimethamine (for the treatment of malaria), as well as large doses of nonsteroidal anti-inflammatory drugs [44]. The 677 C→T (cytosine-thymine) gene polymorphism in methyltetrahydrofolate reductase was found to affect up to 30% of various population groups studied, mainly in developed countries, and the homozygote was associated with increased plasma homocysteine level and low serum and erythrocyte folate levels [56].

Epidemiology of insulin resistance

The worldwide incidence of MS ranges from 10% to 40% depending upon the region and the criteria used to define it [57]. The evolution of MS depends upon several factors such as obesity and insulin resistance [58]. These factors, in turn, result in an increased probability of type 2 diabetes mellitus [58,59]. Furthermore, a strong correlation exists between obesity, insulin resistance, and polycystic ovarian syndrome (PCOS) in women [60].

In the United States alone, the incidence of childhood obesity has nearly tripled from 7% to 18% between 1980 and 2013 [61]. Developing nations have also seen a 4.8% rise in obesity from 1980 to 2013 [62]. In 2014, 13% of the adult population was obese [63]. Concurrently, there were reportedly 422 million insulin-resistant diabetics in the world [64]. These numbers are only expected to escalate in the future and are predicted to increase up to 649 million by 2040 [65]. It is important to note that there has been a significant number of diabetes-related deaths globally (1.6 million) [64]. Type 2 diabetes is also a common side effect of PCOS [66]. Approximately, 60% to 80% of PCOS-affected women suffer from insulin resistance [67].

Pathophysiology of metabolic syndrome/insulin resistance

There is no specific mechanism that leads to the development of MS. Multiple studies have reported that there is an association between insulin resistance and visceral adiposity [68]. Thus, being a state that predisposes to several comorbidities, additional clinical conditions such as hypertension and dyslipidemia may emerge. In addition, it can be seen as a condition in which there is an increment in both thrombotic and inflammatory states distinguished by an elevation in the inflammatory cytokine activity [69].

Insulin resistance is a decrease in the biological function of insulin, irrespective of the serum concentration [70]. Insulin resistance can be taken as the main pathophysiology of MS. Second, the increased prevalence of insulin resistance is related to the global increase in visceral adiposity [71]. Central obesity can emerge from multiple factors, such as an inappropriate diet of consuming higher processed calories, genetic predisposition, or epigenetic factors that can relate to the environment. Recently, a correlation has been reported between the high intake of fats and an inadequate function of the intestinal barrier that can ease the movement of intestinal luminal content and bacterial lipopolysaccharide into the circulation, which throws further light on its pathophysiology [72-74].

One of the main causes that develop this resistance mechanism is the excess of free fatty acids circulating from an expanded adipose tissue mass. This additional fat decreases the insulin sensitivity in the skeletal muscle by inhibiting the glucose uptake mediated by insulin [72]. This also increases the production of endogenous glucose. With the failure of glucose transporter type 4 (GLUT4), which is mainly located in adipocytes and muscle cells, glucose intake is reduced [75-79]. This causes an increase in circulating glucose that leads to increased insulin secretion. Consequently, glucose transporter types 1-3 (GLUT1-3), which are insulin-independent and found in neurons, renal cells, and erythrocytes, are exposed to a large amount of glucose that leads to glucotoxicity [75]. This results in prediabetes, a state characterized by glucose intolerance [80,81]. Additionally, the continuation of insulin resistance can lead the beta cells of the pancreas to get to a point where they can no longer provide the high amounts of insulin secreted before, leading to a drop in insulin secretion and further failure of glucose homeostasis [80,82].

Furthermore, there is an increase in the synthesis of triglycerides and very-low-density lipoproteins (VLDL) that contribute to the failure of the GLUT4 transporter [72,80]. According to the portal theory of MS, there are free fatty acids that are released from the accumulation of visceral fat. They enter the portal circulation and are transported to the liver where they remain as triglycerides [83,84]. This induces the release of VLDL by the liver and causes a state of hypertriglyceridemia [85]. These free fatty acids act on the phosphoinositide-3-kinase activity reducing its function and worsening insulin resistance [80]. These fats also contribute to the reduced production of insulin by the beta cells of the pancreas by exerting a lipotoxic effect on them [77]. Moreover, they contribute to endothelial effect by producing reactive oxygen species, along with the hyperinsulinemia state and the cytokines produced by the adipose tissue [83,86-88].

Regarding MS and inflammation, the state of inflammation in this syndrome is associated with damage of the gut barrier activity and bacterial exposure [89,90]. This endotoxemia may activate inflammatory pathways, along with further encroachment to various organs that can lead to an invasion of macrophages that help promote insulin resistance [91-93]. Additionally, it contributes to the accumulation of adiposity and insulin resistance [94].

There are several ways by which the microbiome may contribute to the development of obesity such as encouraging the accumulation of fat deposits and change in the locomotor activity setting off systemic inflammation [95]. Recently, a correlation has been reported between the high intake of fats and an inadequate function of the intestinal barrier that can ease the movement of intestinal luminal contents and bacterial lipopolysaccharide into the circulation [94,96]. These lipopolysaccharide chain cluster of differentiation 14 (CD14) receptors promote systemic inflammation [97].

Current treatment protocols 

As mentioned above, MS is a multifaceted disease. Therefore, the management approach is targeted toward the prevention and reduction of cardiovascular risk factors such as obesity, type 2 diabetes mellitus, and hypertension [98]. Treatment goals are achieved through various modalities such as lifestyle modification and pharmaceutical management in high-risk individuals [99].

Lifestyle Modifications

To reduce the incidence of MS, early measures such as a low carbohydrate diet, increased physical activity, and controlled alcohol consumption are targeted, in particular, toward the younger generation [100,101]. Lifestyle changes have proven to be the more effective approach to reducing the incidence of MS and diabetes. In the Diabetes Prevention Study conducted over three years, a diabetic case reduction of 41% was seen with lifestyle changes compared to a 17% reduction only with metformin [102]. A diet commonly referred to as “the Mediterranean diet” is efficacious in preventing insulin resistance [103]. A Mediterranean diet consists of a wide range of fruits, vegetables, whole grains, and healthy fats such as seeds, nuts, and extra virgin olive oil. A limited quantity of fish, dairy, poultry, and red meat are also incorporated [104]. The rich plant sources consist of phenolic compounds that modify the insulin activity in the tissue and improve sensitivity [103,105]. Research-based recommendations for diet include 45% to 60% of carbohydrate intake (sourced from legumes and whole grains predominantly), 10% to 20% protein, and 10% fatty acids. [106,107]. High fiber intake (40 g/day) also improves insulin sensitivity [107]. Although regular moderate physical exercise improves MS [108], the best results are observed when exercise is implemented in conjunction with an appropriate diet. A study conducted by Anderssen et al. compared the effects of combined interventions with those without dietary restrictions. They reported a 67% and 24% drop in MS incidence in the combined and isolated groups, respectively [109]. An intervention study, the Da Qing Trial, further supported the abovementioned findings. A more significant reduction of type 2 diabetes mellitus in the combined diet and exercise groups was observed compared to the isolated diet and activity groups (46% vs. 43.8% vs. 41.1%, respectively) [110].

Pharmaceutical Management

A well-established relationship exists between MS and type 2 diabetes mellitus [111], indicating that strict glycemic control can eliminate the risk factors of MS [112]. The two classes of drugs that predominantly affect insulin resistance are biguanides (metformin) and thiazolidinediones (glitazones) [113,114]. Metformin achieves the desired results through various mechanisms such as inhibiting glucose production in the liver [115], increasing glucose reuptake via insulin receptor activation, stimulating tyrosine kinase in the peripheral tissue [113,116], and lowering free fatty acid oxidation in adipose tissues [117]. A study on the mechanism of metformin by Hundal et al. demonstrated a 25% to 30% decrease in plasma glucose because of the actions of metformin on hepatocytes [115].

Glitazones improve metabolic control by binding with nuclear receptor PPAR-γ and controlling glucose metabolism [118]. PPARs are ligand-inducible transcription factors that belong to the nuclear hormone receptors family. Three different isoforms of PPARs have been identified, namely, PPAR-α, PPAR-β/δ, and PPAR-γ. These are involved in adipogenesis, inflammation, lipid, and glucose metabolism [119,120].

Fenofibrate, the prototype of fibrate drugs, activates PPAR-α whereas bezafibrate is a pan-PPAR activator for all three PPAR isoforms. In a recent meta-analysis by Simental-Mendía et al., fibrate therapy significantly reduced fasting plasma glucose (-0.28 mmol/L), insulin levels (-3.87 pmol/L), and insulin resistance [Homeostasis Model of Assessment for Insulin Resistance (HOMA-IR) -1.09], with no effect on glycated hemoglobin (HbA1c) [121]. These effects are more pronounced with bezafibrate as it is a pan-PPAR activator.

All three drug classes have been hypothesized to have a protective effect on the cardiovascular system [122-124]. However, these results are debatable and require further investigation.

Several other targeted therapies are implemented with the predominant metabolic risk factor in mind. In severe hypertension, newly introduced angiotensin receptor blockers (telmisartan and irbesartan) have shown promising results in comparison to other antihypertensive medications. These drugs behave similarly to glitazones and stimulate PPAR-γ, resulting in a reduction of blood pressure and an increase in insulin sensitivity [125].

In severe obesity (a BMI of at least 30 kg/m^2^), where all other treatments have been unsatisfactory, pharmacological management can be considered [126]. The current treatment options include sibutramine and orlistat. A meta-analysis of randomized trials on sibutramine showed a 4.6% more weight loss than the placebo [127]. Increased insulin sensitivity and better glycemic control were also reported among diabetics [128]. In a diabetic study [129], a combined orlistat and diet regimen reduced MS by 35% versus 9% (diet alone). In addition to weight loss, orlistat also prevents type 2 diabetes mellitus and hyperglycemia [130].

Statins are primarily used as LDL-C-lowering agents in patients who meet the criteria for atherosclerotic cardiovascular disease (ASCVD) with MS. Its pleiotropic effects have proven to be significantly beneficial in patients with MS in reducing major coronary events [2,98]. MS has been associated with elevated C-reactive protein (CRP); statins lower CRP and reduce pro-inflammatory states, further exhibiting their prognostic value [98,99]. An interventional study in patients with MS focused on measuring effective reductions in cholesterol using rosuvastatin therapy (MERCURY Trial) found 10 mg of rosuvastatin to be more promising compared to commonly used doses of other statins in achieving the target LDL-cholesterol level [98]. A similar study conducted by Grundy et al., the Comparative study with Rosuvastatin in Subjects with Metabolic Syndrome (COMETS), also demonstrated rosuvastatin to be an effective lipid-modifying agent in patients with MS. Moreover, recent studies have established that the combination of statins and fibrates or nicotinic acid is more efficient in reducing cardiovascular outcomes in addition to minimizing the risks of myopathy [99].

Effectiveness of folate and vitamin B12 supplementation in insulin resistance and metabolic syndrome

Folate supplementation has been established with protective studies in reducing insulin and HOMA-IR; however, inconsistent results have been reported regarding the reduction of fasting blood sugar and HbA1c levels [131]. The dosage of folate supplementation plays an important role in the reduction of insulin resistance [132]. The glycemic markers were effectively reduced with a folate dose greater than 5 mg/day [132]. A systematic review published by Xie et al. in 2016 showed that folic acid supplementation in maternal and prenatal animal groups decreased both insulin resistance and obesity significantly [133]. Further, folate prevented the decline in cognitive skills associated with metformin use in diabetes [134]. To support this, we reviewed a clinical study conducted by Porter et al. (2019) in a group of community-dwelling older adults and found that using fortified vitamin B12 foods improved the patient’s cognitive status [134]. Scientific studies have evolved, paving the way for newer and promising treatments for insulin resistance. One such study used folate nanoparticles in rats to achieve an effective glycemic index [135]. It was demonstrated that using folate nanoparticles showed a five-fold enhancement in cellular uptake of insulin and that blood glucose levels were controlled effectively [135].

Furthermore, it was evident that dietary intake of folate and vitamin B12 had a positive impact on MS and reduced the risk of stroke in both men and women in the United States [136,137]. Spence et al. (2016) showed that supplementation with methylcobalamin minimized the incidence of stroke in persons with renal impairment and cyanocobalamin in patients without any renal impairment [138]. To highlight the relationship between low supplementation of folate and vitamin B12 and stroke, we reviewed a randomized controlled trial (RCT) conducted by Qin et al. (2020) in a double-blinded hypertensive population. The study revealed that people with low levels of folate and vitamin B12 were associated with an increased risk of ischemic stroke [139]. The duration of folate supplementation influenced the incidence of stroke by a reduction of 18% in people who consumed it for more than 36 months [140].

Another area of interest was to assess the effect of folate/vitamin B12 on cardiovascular diseases. Studies have highlighted plasma homocysteine levels to play an important role in the occurrence of myocardial infarction, and its levels were predominantly dependent on plasma folate values to a greater extent and vitamin B12 to a lesser extent [141]. This was further supported by a prospective study conducted by Stampfer et al. (1992) which assessed the risk of plasma homocysteine and cardiovascular diseases in U.S. physicians and found that the levels of homocysteine were higher in cases than in controls (11.1 ± 4.0 standard deviation [SD] vs. 10.5 ± 2.8 nmol/mL) who later developed myocardial infarction, irrespective of other modifiable risk factors [142]. Furthermore, low vitamin B12 levels have been associated with the development of atherosclerosis, which is an important causative factor for cardiovascular diseases [143]. Moreover, because hyperhomocysteinemia has been linked with congenital heart disease malformation in children, periconceptional folate supplementation has proven to be a key factor in reducing congenital heart disease in children [144].

Along with cardiovascular diseases and stroke, another common condition associated with low folate/vitamin B12 levels is obesity [145]. A study by Kumar et al. (2012) included female Wistar rats which were deprived of a vitamin B12 diet and allowed to mate [146]. Biochemical and body parameters were measured in maternal rats before mating and in offspring at three, six, nine, and twelve months of age. It was demonstrated that the maternal rats suffered from increased body fat and altered lipid profile and the offspring had an increase in fatty acid synthase and higher plasma cortisol levels, thus paving the way to high visceral fat at three months and dyslipidemia at 12 months [146]. Therefore, it is recommended that pregnant females remain careful about their folate/vitamin B12 supplementation to prevent dyslipidemia and neural tube defects in the offspring [147]. It has been reported that both folate and vitamin B12 have a synergistic effect on the markers of obesity and lipid profile, such as the waist to hip length ratio, triglyceride level, and HDL-C level [148-150]. However, further clinical studies are required to warrant any unwanted side effects with the use of folate and vitamin B12 for insulin resistance and MS.

Novel therapeutic approaches and targets

MS is a constellation of disorders that usually involves the treatment of the individual disorder, along with dietary intervention and physical activity [98]. Due to a better understanding of the pathophysiology of MS, novel agents acting multiple targets and mechanisms are being developed [151].

Role of Selective Peroxisome Proliferator-Activated Receptor Modulators 

Novel compounds, such as selective peroxisome proliferator-activated receptor modulators (SPPARMs), have more potent PPAR-α agonist activity. These are proven to have advantages in the treatment of insulin resistance and dyslipidemia. One such agent is pemafibrate. Pemafibrate is a novel, highly selective PPAR-α agonist approved for the treatment of hyperlipidemia in Japan. The recommended dosage of oral pemafibrate is 0.1 mg twice daily, which can be adjusted to a maximum of 0.2 mg twice daily [152]. It is more robust in reducing triglycerides and elevating HDL-C, thereby enhancing reverse cholesterol transport compared to fenofibrate [153]. Based on studies in mouse models, pemafibrate is more efficient in decreasing apolipoprotein B-48 (ApoB-48)-containing chylomicron remnants than those containing ApoB-100. It is noteworthy that ApoB-48 is more atherogenic [154]. In an RCT conducted by Araki et al. on type 2 diabetes mellitus patients (HbA1c ≥ 6.2%) with fasting triglycerides of ≥150 mg/dL, not on statins, participants were randomly assigned to pemafibrate at doses of 0.2 mg/day, 0.4 mg/day, and placebo. After 24 weeks of treatment, triglycerides declined by 45% in both doses; fasting triglycerides of ≤150 mg/dL were achieved in 70.9% and 81.5% of patients on 0.4 mg/day and 0.2 mg/day, respectively. No considerable changes in LDL-C were noted. However, an increment in HDL-C and ApoA-I was observed [155]. Among glycemic parameters, a significant decrease in HOMA-IR was noticed in the 0.2 mg/day pemafibrate group, while no significant changes were noted in other parameters. The most common adverse effects reported were increased serum creatinine and liver enzymes [155].

The most recent evidence regarding pemafibrate is yet to be obtained through a Pemafibrate to Reduce Cardiovascular OutcoMes by Reducing Triglycerides IN patiENts With diabeTes (PROMINENT) study which is a multicentre, randomized, phase 3 trial. The study recruited approximately 10,000 participants from 24 countries with type 2 diabetes mellitus, mild-to-moderate hypertriglyceridemia, and low HDL-C levels to either pemafibrate (0.2 mg twice daily) or matching placebo. The trial is currently ongoing with a mean follow-up period of 3.75 years [156]. Further studies are needed to determine the effect of pemafibrate in preventing cardiovascular disease.

Farnesoid X Receptor Agonists

Farnesoid X receptor (FXR) belongs to the nuclear receptor family. Among bile acids, chenodeoxycholic acid is the most potent activator of FXR. These receptors are expressed in the liver, kidney, intestine, and adrenal glands [157]. Activation of FXR has been shown to increase insulin sensitivity and decrease triglycerides and free fatty acid levels. Recent data also suggest reduced endothelin levels, thereby playing a role in atherosclerosis [157]. Recently, obeticholic acid, an FXR agonist, has been approved for the treatment of primary biliary cholangitis. Newer FXR agonists such as nidufexor or tropifexor are under phase 2 clinical trials for the treatment of nonalcoholic fatty liver disease and non-alcoholic steatohepatitis, considered to be a hepatic manifestation of MS [158,159].

Asprosin and Asprosin Neutralizing Antibodies

Asprosin, a glucogenic adipokine, was discovered in 2016 in the study of the neonatal progeroid syndrome [160]. Sites of action include the liver and the brain. In the liver, it exerts a glucogenic effect through the cyclic adenosine monophosphate protein kinase A (cAMP-PKA)-dependent pathway [161]. It crosses the blood-brain barrier and stimulates appetite by activating orexigenic and inhibiting anorexigenic neurons in the arcuate nucleus of the hypothalamus. The levels have been found to be elevated in MS [161,162].

A preclinical study done on mice by Mishra et al. showed that a single dose of an anti-asprosin monoclonal antibody (mAb) reduced food intake by an average of 1 g/day, resulting in decreased body weight. Other parameters such as triglycerides, blood glucose, and plasma insulin levels also decreased [162]. Anti-asprosin mAbs also exert action at the target level by inhibiting hepatic cAMP signaling and orexigenic neuron activity in the hypothalamus, in addition to neutralizing asprosin [161,162]. Further studies are required in humans to delineate efficacy and safety, and these preclinical study results offer better hope for the future.

Peripheral Cannabinoid Receptor Antagonists

Selective cannabinoid (CB) receptor inhibition is a novel approach to treat MS and dyslipidemias. Cannabinoid receptors CB-1 and CB-2 are G protein-coupled receptors that activate G proteins (Gi/Go). CB-1 receptors are expressed in the brain and peripherally in the heart, liver, pancreas, and adipose tissue, whereas CB-2 receptors are found in immune cells [163]. Although the exact mechanism of CB receptor antagonists is not well understood, they have been found to modulate leptin levels. Another mechanism is through the activation of the peripheral sympathetic system [164].

The use of the CB-1 receptor antagonist dates back to 2006. Rimonabant, approved for the treatment of obesity, was withdrawn in 2008 because of an increase in depressive mood disorders associated with antagonism of central CB-1 receptors [165]. This leads to the development of compounds with limited central nervous system penetration and action on peripheral CB receptors. However, further studies are needed to demonstrate relevant metabolic endpoints [166]. Newer compounds of the CB receptor family which are in the preclinical phases of investigation are showing promising results [167,168].

Role of Fecal Microbiota Transplantation in Insulin Resistance

The human gut microbiome harbors an estimated 10-100 trillion microorganisms. Gut microbial dysbiosis has been defined as alterations in diversity with more “inflammatory” microbes (i.e., Proteobacteria) and decreased short-chain fatty acid levels [169].

A recent study among 154 human twins shed light on the link between obesity and gut microbial diversity. Excess Bacteroidetes and deficient butyrate and propionate levels were noted in the gut microbiome of twins with obesity phenotype [170,171]. Two randomized control trials conducted by Kootte et al. and Vrieze et al. showed similar effects, such as improvement in insulin sensitivity and increased microbial diversity at six weeks after allogenic fecal microbiota transplantation (FMT) compared to autologous FMT [171,172]. Further, when metabolic parameters were measured at 18 weeks in the study by Kootte et al., no changes were observed. This study showed that the short-term effects of FMT are not maintained in the long term [171].

Although the role of FMT in insulin resistance is recognized, the role of FMT in hypertension is unclear. In studies on rat models for hypertension, a decreased microbiota diversity in the form of decreased Firmicutes/Bacteroidetes ratio was observed. Interestingly, the blood pressure in these rat models could be controlled by reversing the Firmicutes/Bacteroidetes ratio by utilizing antibiotics [173,174]. More controlled trials are needed to elucidate the potential benefit and sustainability of FMT in MS.

Adenosine Triphosphate Citrate Lyase Inhibitors

Adenosine triphosphate citrate lyase (ACLY) is a cytoplasmic enzyme that mediates the production of acetyl coenzyme A (acetyl-CoA) that acts as a substrate for the de novo synthesis of fatty acids and cholesterol [175].

Bempedoic acid, an ACLY inhibitor, is approved by the U.S. Food and Drug Administration (US FDA) for the treatment of heterozygous familial hypercholesterolemia (HeFH) and established ASCVD [175]. Bempedoic acid modulates adenosine monophosphate-activated protein kinase (AMPK) activation and decreases hepatic glucose production, an action similar to metformin [176]. In a randomized, 52-week, phase 3 trial, involving adults with ASCVD, HeFH, or both, bempedoic acid at a dose of 180 mg once daily reduced the mean LDL-C level by 12.6% by week 52 [177]. The most frequent adverse effects observed included nasopharyngitis, muscle spasms, hyperuricemia, anemia, and elevated transaminases [177,178]. ACLY inhibitors can be used as the primary drug in patients who are intolerant to statins. Further studies are needed to evaluate the role of ACLY inhibitors in reducing inflammation, insulin resistance, and other components of MS.

Central Leptin Gene Therapy

The role of leptin resistance in MS has long been proven. Leptin, an adipogenic hormone, acts on the hypothalamus and increases satiety [179]. Burguera et al. postulated that “leptin resistance might be due to non-availability of leptin at hypothalamic target sites caused by either defective uptake across the blood-brain barrier, or abnormal post-receptor signal transduction” [180].

Using recombinant adeno-associated virus vector for central leptin gene therapy in animal models showed decreased weight, insulin resistance, and increased thermogenesis [181]. Further long-term studies are required in humans as leptin gene therapy can offer a permanent solution to multiple components of MS.

## Conclusions

This review aimed to understand the interactions between MS and vitamins B12 and folate and summarized the most relevant literature. To date, MS remains somewhat of an enigma as there is no consensus on how or why it develops. However, progress has been made on the therapeutic end, with a whole host of novel drugs being developed to treat the condition. Moreover, the impact of folate and vitamin B12 supplementation is a new frontier that is still relatively unexplored. We recommend further observational studies and RCTs that examine exactly how much vitamin supplementation affects each component of MS. Current literature suggests that these vitamins have a significant positive impact on MS, and they must be kept in mind while designing new treatment protocols in the future.
